# The Detailed Pharmacodynamics of the Gut Relaxant Effect and GC-MS Analysis of the *Grewia tenax* Fruit Extract: In Vivo and Ex Vivo Approach

**DOI:** 10.3390/molecules27248880

**Published:** 2022-12-14

**Authors:** Najeeb Ur Rehman, Mohd Nazam Ansari, Wasim Ahmad, Mohd Amir

**Affiliations:** 1 Department of Pharmacology & Toxicology, College of Pharmacy, Prince Sattam Bin Abdulaziz University, Al-Kharj 11942, Saudi Arabia; 2 Department of Pharmacy, Mohammed Al-Mana College for Medical Sciences, Dammam 34222, Saudi Arabia; 3 Department of Natural Products and Alternative Medicine, College of Clinical Pharmacy, Imam Abdulrahman Bin Faisal University, Dammam 31441, Saudi Arabia

**Keywords:** *G*. *tenax*, GC-MS, anti-spasmodic, Ca^2++^ channel blockers, phosphodiesterase

## Abstract

The study was performed to assess and rationalize the traditional utilization of the fruit part of *Grewia tenax* (*G*. *tenax*). The phytoconstituents present in the methanolic extract were analyzed using Gas-Chromatography-Mass Spectroscopy (GC-MS), while the anti-diarrheal activity was investigated in the Swiss albino mice against castor oil-provoked diarrhea in vivo. The antispasmodic effect and the possible pharmacodynamics of the observed antispasmodic effect were determined in an isolated rat ileum using the organ bath setup as an ex vivo model. GC-MS findings indicate that *G*. *tenax* is rich in alcohol (6,6-dideutero-nonen-1-ol-3) as the main constituent (20.98%), while 3-Deoxy-d-mannoic lactone (15.36%) was detected as the second major constituents whereas methyl furfural, pyranone, carboxylic acid, vitamin E, fatty acid ester, hydrocarbon, steroids, sesquiterpenes, phytosterols, and ketones were verified as added constituents in the methanolic extract. In mice, the orally administered *G*. *tenax* inhibited the diarrheal episodes significantly (*p* < 0.05) at 200 mg/kg (40% protection), and this protection was escalated to 80% with the next higher dose of 400 mg/kg. Loperamide (10 mg/kg), a positive control drug, imparted 100% protection, whereas no protection was shown by saline. In isolated rat ileum, *G*. *tenax* completely inhibited the carbamylcholine (CCh; 1 µM) and KCl (high K^+^; 80 mM)-evoked spasms in a concentrations-mediated manner (0.03 to 3 mg/mL) by expressing equal potencies (*p* > 0.05) against both types of evoked spasms, similar to papaverine, having dual inhibitory actions at phosphodiesterase enzyme (PDE) and Ca^2+^ channels (CCB). Similar to papaverine, the inhibitory effect of *G*. *tenax* on PDE was further confirmed indirectly when *G*. *tenax* (0.1 and 0.3 mg/mL) preincubated ileal tissues shifted the isoprenaline-relaxation curve towards the left. Whereas, pre-incubating the tissue with 0.3 and 1 mg/mL of *G*. *tenax* established the CCB-like effect by non-specific inhibition of CaCl_2_–mediated concentration-response curves towards the right with suppression of the maximum peaks, similar to verapamil, a standard CCB. Thus, the present investigation revealed the phytochemical constituents and explored the detailed pharmacodynamic basis for the curative use of *G*. *tenax* in diarrhea and hyperactive gut motility disorders.

## 1. Introduction

Both diarrhea and hyperactive gut are gastrointestinal motility disorders responsible for morbidity and mortality, especially in developing countries [1]. Diarrhea is characterized by three or more episodes of defecation with unusually loose and watery stools. The severity of the diarrhea is scored based on its frequentness, constancy, and mass of the stool [2]. It is usually associated with gastric pain, fecal urgency, perianal discomfort, and bowel incontinence [3]. Diarrhea is not a disease in and of itself but rather a sign and symptom caused by a combination of factors [4,5].

Diarrhea is both preventable and curable. Its treatment is non-specific and is more focused on decreasing the discomfort and inconvenience of frequent bowel motions. Even with a wide range of interventions for the management of diarrhea, three-quarters of the population in developing countries still depend on traditional medicine for essential health care, including the management of diarrhea [6]. The currently used drugs on the market to manage diarrhea have limitations associated with their adverse effects and contraindications. For instance, the occurrence of vomiting, bronchospasm, and fever is associated with the administration of racecadotril and loperamide (used to cure secretory diarrhea). Additionally, loperamide is reported to cause intestinal obstruction in children below 6 years of age and hence is contraindicated in children [7].

A major proportion of the population in underdeveloped and emergent nations relies on the utilization of traditional medicines for the treatment of several disease conditions, including diarrhea [8,9]. Hence, the search for a plant with promising antidiarrheal potential draws lots of attention from researchers. In the traditional system of medicine, multiple medicinal plants have been reported for antidiarrheal potential. However, many of these plants are not backed by scientific evaluation. As a result, scientific research exploring the curative prospects of these herbs is critical to search for new anti-diarrheal agents with distinctive mode(s) of action and with synergistic and/or side-effect neutralizing components.

The genus Grewia (family: Tiliaceae) is distributed in the tropics and sub-tropics globally and contains approximately 150 species. It is also the only member of the family that produces eatable fruits [10]. *Grewia tenax* (*Forsk*.) *Fiori* “Guddaim” is a largely valued wild shrub in Sudan, particularly in the Northern and Middle Sudan, majorly distributed in arid and semi-arid zones [11]. The fruit (per 100 g) has a substantial amount of potassium (817 mg) and iron (20.8–22.3 mg), and hence it is given as an iron supplement to treat anemia in children. Compared to other *Grewia* spp., *G*. *tenax* contains a significant amount of reducing sugar (13.8%) and starch (44.4%) [12]. The composition of *G*. *tenax* fruit in % is D-fructose (24.3), D-glucose (21.0), starch (15.1), fiber (8.1), protein (6.3), ash (4.5), sucrose (1.6), and fat (0.4) [13]. *G*. *tenax* fruits are predominant in sugars, starch, tannins, phenolic compounds, flavonoids, steroids, vitamin C, and protein [14,15].

The fruits are sweet and can be consumed whole, in parts, or as juice. Traditionally, the Guddaim juice is usually indicated for breastfeeding women as it promotes their health and lactation. It is considered a good iron source for children less than 8 years of age [16]. It is extensively used for curing several common illnesses, such as diarrhea, dysentery, cough, fever, jaundice, and rheumatism [17]. The seeds and the green leaves are utilized during the animal delivery season [18]. The powder of the fruit is mixed with the milk and is used to cure fractures and bone swelling [19].

Despite multiple reported activities such as antimicrobial, antibacterial, antifungal [20], hepatoprotective and antimalarial [21], to date, to the best of our knowledge, the antidiarrheal and antispasmodic potential of *G*. *tenax* fruit with detailed mechanistic has not been evaluated. Consequently, the study is directed to evaluate the anti-diarrheal and anti-spasmodic potential of the *G*. *tenax* extract using ex vivo and in vivo experimental models, whereas the phytochemical composition was analyzed using the GC-MS technique.

## 2. Results

### 2.1. Methanolic Extract Yield (%)

The finally concentrated methanolic extract yield of *G*. *tenax* fruit was found to be 25.97%.

### 2.2. GC-MS Phytochemical Profiling

**Figure 1:**
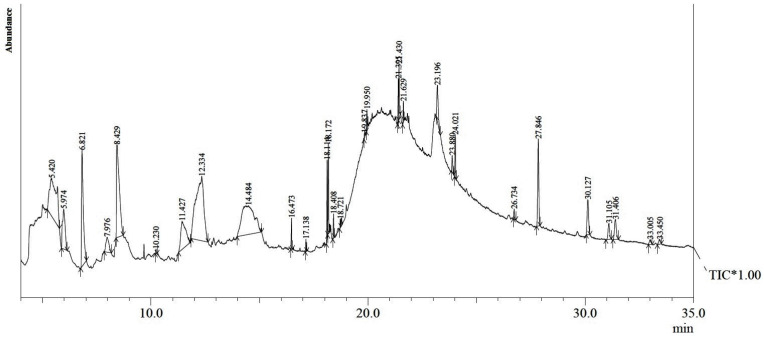
GC-MS total ion chromatogram of *G*. *tenax* fruits methanolic extract.

Table 1 presents the detailed phytoconstituents of *G*. *tenax* fruit, identified by GC-MS. A total of -28 constituents were identified. A typical GC-MS chromatogram is shown in Figure 1. Alcohol (6,6-dideutero-nonen-1-ol-3) was recorded as the main constituent of the methanolic extract (20.98%), while 3-Deoxy-d-mannoic lactone was recorded as the second most abundant occurring constituent (15.36%). Additionally, methyl furfural, pyranone, carboxylic acid, vitamin E, fatty acid ester, hydrocarbon, steroids, sesquiterpenes, phytosterols, and ketones were verified as other additional constituents in the extract. Further details are available in Table 1 and Figure 1.

### 2.3. In Vivo Antidiarrheal Effect

Oral administration of *G*. *tenax* extract at both lower and higher doses in mice imparted marked protection against castor oil-evoked copious diarrhea as compared to the animals of the saline group (Table 2). The mice treated with a lower dose of 200 mg/kg showed 40% protection as two out of five mice did not drop loose and watery stools, while the next higher dose of 400 mg/kg produced 80% protection, whereas the mice treated with loperamide (10 mg/kg) did not show any spots in their cages, suggesting 100% protection against diarrhea (Table 2).

### 2.4. Ex Vivo Antispasmodic Effects

**Figure 2:**
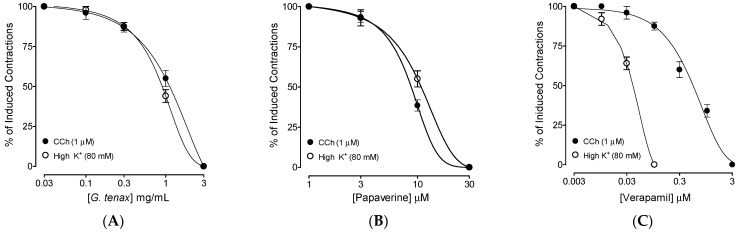
Concentration-response curves presenting a comparison of the inhibitory effect of (**A**) methanolic extract of *Grewia tenax* fruits (*G*. *tenax*), (**B**) papaverine, and (**C**) verapamil against carbachol (CCh; 1 µM) and high K^+^ (80 mM)-evoked contractions in isolated rat ileum preparations. Values are presented as mean ± SEM, *n* = 4.

*G*. *tenax* resulted in complete relaxation of both types of CCh and high K^+^ mediated spasms in isolated rat ileum, with resultant EC_50_ values of 1.22 mg/mL (0.98–1.58, 95% CI, *n* = 4) and 1.34 mg/mL (1.04–1.36, 95% CI, *n* = 4) respectively (Figure 2A). Similarly, comparable relaxant effects were shown by the standard drug, papaverine, with resultant EC_50_ values 8.28 µM (7.86–8.94, 95% CI, *n* = 4) and 8.64 µM (8.12–8.72, 95% CI, *n* = 4), (Figure 2B). On the other hand, verapamil showed significantly higher potency against high K^+^ compared to CCh-mediated spasm, with EC_50_ values of 0.18 µM (0.14–0.21, 95% CI, *n* = 4) and 2.42 µM (2.14–2.85, 95% CI, *n* = 4), respectively (Figure 2C).

### 2.5. Phosphodiesterase Enzyme (PDE) Inhibitory-like Effect

**Figure 3:**
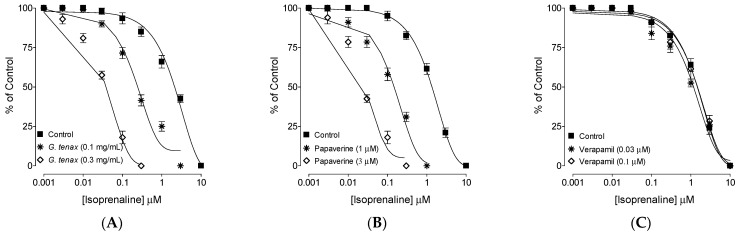
Inhibitory concentration-response curve of isoprenaline against carbachol (CCh)-induced contraction in the absence and presence of various concentrations of (**A**) methanolic extract of *Grewia tenax* fruit (*G*. *tenax*), (**B**) papaverine, and (**C**) verapamil in isolated rat ileum preparations. Values are expressed as mean ± SEM, *n* = 4.

The possible PDE-inhibitory effect of the extract was established by pre-treating the tissue with *G*. *tenax* (0.1 and 0.3 mg/mL), which resulted in the shift of isoprenaline-induced inhibitory CRCs towards the left (Figure 3A), thus depicting its potentiating effect. With the positive control drug, papaverine (1 and 3 µM) similar leftward isoprenaline curve shift was observed (Figure 3B), whereas verapamil did not show a potentiating effect (Figure 3C).

### 2.6. Calcium Channel Blocking (CCB)-like Effect

**Figure 4:**
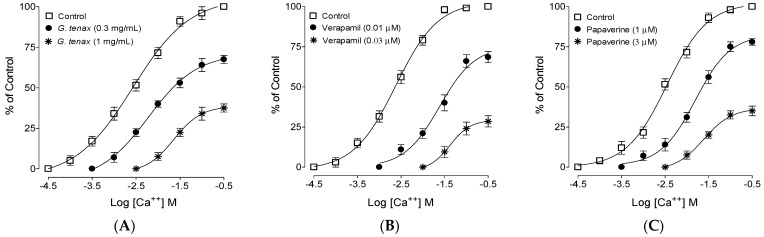
Concentration-response curves of Ca^2+^ in the absence and presence of the increasing concentrations of the (**A**) methanolic extract of *Grewia tenax* fruits (*G*. *tenax*), (**B**) verapamil, and (**C**) papaverine in isolated rat ileum preparations. Values shown are mean ± SEM, *n* = 5.

The Ca^2+^ inhibitory effect of *G*. *tenax* methanolic extract was confirmed by preincubating ileum tissue with extract (0.3 and 1 mg/mL), which resulted in Ca^2+^ CRCs curve shifts towards the right with suppression of the maximum response (Figure 4A), similar to verapamil (0.01 and 0.03 µM; Figure 4B). Papaverine (1 and 3 µM) also displaced the Ca^2+^ curve towards the right, as shown in Figure 4C.

## 3. Discussion

The present study aimed to test the traditional claim of the plant extract of *G*. *tenax* in diarrhea and hyperactive gut disorders and to further explore the detailed pharmacodynamics for the observed antispasmodic effect in rodents, whereas the phytochemical constituents were determined using the GC-MS technique. The extract of *G*. *tenax* was tested against castor-oil-evoked diarrhea, where it showed a dose-mediated antidiarrheal effect by inhibiting diarrheal drops, compared to the animals of the saline-treated control group in which copious diarrhea was observed in all the animals. Castor oil-induced diarrhea model is a well-established assay to test the unknown samples for antidiarrheal potential where it evokes diarrhea in animals as it gets hydrolyzed in the gut into ricinoleic acid, causing severe irritation in the bowel and thus leading to spasms in the gut [22]. Pre-treatment of the *G*. *tenax* extract in mice protected them against diarrhea in a dose-mediated manner similar to loperamide, a positive control drug. For evaluation of possible pharmacodynamics involved in the observed diarrhea protection, the methanolic extract of *G*. *tenax* in cumulative concentration was assessed in isolated rat ileum, considered a standard assay for screening of antispasmodic effect [23]. Based on our previously reported studies, we observed that antispasmodic drugs usually act by blocking Ca^2+^ channels [24,25] and/or PDE inhibition [26]. Based on these reports, we tested *G*. *tenax* extract in rat ileum against CCh and high K^+^-induced contractions [27]. Interestingly, *G*. *tenax* inhibited both types of contraction with comparable inhibitory CRCs patterns against CCh and high K^+^.

Likewise, papaverine, a dual inhibitor of Ca^2+^ channels and PDE-enzyme [28], also inhibited CCh and high K^+^ mediated spasms at comparable concentrations, whereas verapamil, a standard CCB [29,30], selectively showed increased potency against high K^+^ compared to CCh, a typical characteristic of Ca^2+^ channel blocker. Therefore, like papaverine, *G*. *tenax* exhibits dual inhibitory activity against PDE and Ca^2+^ channels. The PDE-inhibitory-like effect of *G*. *tenax* was further authenticated indirectly when the pre-incubated ileal tissues with increasing concentrations of *G*. *tenax* deflected and potentiated the isoprenaline-mediated CRCs towards the left, and this potentiation is most probably considered the resulted increase in cAMP levels in the tissue [31] Phosphodiesterase enzymes converts cAMP (active form) to AMP (inactive form); thus any substance that has an inhibitory effect on PDE will cause an increase in cAMP and will thus produce relaxation of smooth muscle [32]. A similar type of potentiation of inhibitory CRCs of isoprenaline towards lower doses (leftward) was seen in pre-incubated tissues with papaverine, a standard PDE inhibitor [31]. Our current findings are further supported by the previous studies that reported CCh-mediated smooth muscle spasms inhibited by PDE inhibitors [33,34].

High K^+^ (>30 mM) produces contraction by depolarizing the tissue via activation of voltage-gated L-type Ca^2+^ channels, and a substance that reverses this contraction is known to possess CCBs-like activity [35]. Hence, the CCB-like action of *G*. *tenax* was assumed when it inhibited K^+^-80 mM-induced contractions, and this CCB-like effect was further confirmed when preincubation of ileum tissues with increasing concentrations of the extract in Ca^2+^ free tissues caused rightward deflection of Ca^2+^-CRC with suppression of the maximum peak similar to verapamil, a standard CCB, thus confirming the CCB-like effect. The phytoconstituents of *G*. *tenax* fruit methanolic extract identified by GC-MS show 28 constituents, and their % yield showed alcohol (6,6-dideutero-nonen-1-ol-3) as the main constituent of the methanolic extract (20.98%), whereas, 3-Deoxy-d-mannoic lactone (15.36%) was recognized as the next major constituents. Methyl furfural, pyranone, carboxylic acid, vitamin E, fatty acid ester, hydrocarbons, steroids, sesquiterpenes, phytosterols, and ketones were verified as other constituents in the extract.

## 4. Materials and Methods

### 4.1. Extraction of Plant Material

The fruits of *G*. *tenax* were collected from the local market of Dammam (Saudi Arabia) and authenticated with a voucher specimen of PL/045/2020-21/P-011 by the Department of Pharmacognosy, College of Clinical Pharmacy, Taif University, Saudi Arabia. A total of 40 gm of dried fruit powder was extracted using a soxhlet unit using 200 mL of methanol, and the concentrated extra was kept at 5–10 °C in an airtight glass container until further usage.

The % yield of the extract was calculated using the given formula. The reported methods were used for phytochemical investigations of the extract [36,37].

$$\%\;\mathrm{Extraction}\;\mathrm{yield}=\frac{\mathrm{Weight}\;\mathrm{of}\;\mathrm{dried}\;\mathrm{extract}}{\mathrm{Weight}\;\mathrm{of}\;\mathrm{drug}\;\mathrm{sample}}\times 100$$

### 4.2. Chemicals

Carbamylcholine (carbachol; CCh), loperamide, acetylcholine perchlorate (Ach), isoprenaline, verapamil, and papaverine were obtained from Sigma Company, St. Louis, MO, USA. Salts for a physiological salt solution such as potassium chloride (Sigma Co.), calcium chloride, glucose, magnesium sulfate, potassium dihydrogen phosphate, sodium bicarbonate, and sodium chloride were obtained from Merck, Germany. All the chemicals were of analytical grade. Castor oil was procured from a nearby pharmacy.

### 4.3. Animals

Rats (200–250 g) for ex-vivo and Swiss albino mice (30–35 g) for in vivo experiments were procured from the Animal Care Unit, College of Pharmacy, Prince Sattam bin Abdulaziz University, Saudi Arabia. They were kept in optimum conditions of temperature (22 ± 1 °C), relative humidity (55 ± 5 °C), and balanced light/dark cycle exposure. All the animals were fed, including a standard pellet diet and water *ad libitum*. Experimental animals, i.e., rats, were kept on fasting for 24 h before performing ex vivo experiments, and cervical dislocation was used after light anesthesia. The NRC instructions were followed while conducting the experiments on animals [38]. The approval of the study protocol was obtained by the Bio-Ethical Research Committee (BERC) at Prince Sattam Bin Abdulaziz University with the reference number BERC-004-12-19.

### 4.4. GC-MS Analysis

Gas-chromatography mass spectroscopy technique was employed for the phytochemical investigation of the methanolic extract of *G*. *tenax*. Phytoconstituents separation was attained on Agilent GCMS (Agilent Technologies, Santa Clara, CA, USA) the capillary column 60 M TRX 5-MS (30 m × 250 µm film) by utilizing a 2 µL aliquot of sample. The oven temperature program was as follows: 80 °C initially for 3 min and then ramped at a rate of 10 °C/min to 280 °C for 19 min. Helium was used as a carrier gas with a flow rate of 1.21 mL/min. The set temperature for the injector and source was 260 °C and 220 °C, respectively. The electron ionization energy system was employed with 70 eV at a multiplier voltage of 380 within the *m/z* range.

Individual phytoconstituents were identified by referring to the Institute of Standards and Technologies (NIST) libraries [37,39].

### 4.5. In Vivo Antidiarrheal Study

The assessment of the possible potential of the extract to protect mice from diarrhea was done according to the previously described method with a slight modification [40]. A random grouping of twenty mice was followed with an equal number of mice into four groups, and the mice were kept on fasting for 24 h. The Group 1 mice were treated with normal saline (10 mL/kg, oral) and categorized as a negative control group. The mice of the tested group 2 and 3 were administered with the 2 increasing doses of *G*. *tenax* 200 and 400 mg/kg, respectively. On the basis of an acute toxicity test conducted in mice, *G*. *tenax* extract was found safe at 4.0 g/kg, and thus 1/20th and 1/10th (200 and 400 mg/kg, respectively) were chosen for the possible antidiarrheal effect. Group 4 was categorized as a positive control group and administered 10 mg/mL of loperamide. Each mouse was placed in a separate cage with a blotting paper placed at the base of the cage to identify and mark the absence and presence of diarrhea by a blind observer. All the experimental animals were administered 10 mL/kg of castor oil orally, using a 1 mL syringe after 1 h for the respective groups of saline, extract, and loperamide treatment. All the blotting papers in each cage were examined after 4 h for typical diarrheal dropping. The protection was observed if the blotting paper lacked the diarrheal drops, as reported by Rehman et al. [41].

### 4.6. Ex Vivo Experiments on Isolated Rat Ileum

The rats were sacrificed, and their ileum was isolated as per the method of Rehman et al. [42]. Further, 2–3 cm ileum tissue segments were freed from adjoining tissues and fecal material. The mounting of the ileum was then done with emkaBath (France), fitted with a transducer and IOX software. The tissue bath (20 mL) was filled with fresh tyrode solution and gassed with carbogen while the temperature was maintained at 37 °C. The composition of the concentration (mM) of Tyrode’s solution was as follows: NaCl 136.9, KCl 2.68, MgCl_2_ 1.05, NaHCO_3_ 11.90, CaCl_2_ 1.8, NaH_2_PO_4_ 0.42, and Glucose 5.55 (pH 7.4). To apply a tension of 1 g to the tissue, the transducer knob was rotated clockwise, and for stabilization, it was left for 30 min with several exposures of acetylcholine (0.3 µM). After stabilization, the tissue was exposed to spasmogens; CCh and high K^+^ to evoke sustained contraction. Afterward, the *G*. *tenax* and standard drugs, from low to higher concentrations, were added to the bath solution till complete inhibition was observed. Once the tested samples’ relaxant effects against CCh and high K^+^-mediated contractions were found, further experiments were conducted to explore the detailed mechanism(s) of *G*. *tenax* on inhibitory effects on voltage-gated Ca^2+^ channels and/or PDE inhibition by following the earlier assays reported by Godfrained et al. [43]. When multiple smooth muscles are exposed to K^+^ (>30 mM), it will cause activation of the L-type C^++^ channel, leading to depolarization, and hence sustained contractions are observed in smooth muscles. Moreover, any test material that will inhibit sustained contraction induced by CCh and high K^+^ with similar potencies is labeled as a PDE inhibitor [44].

### 4.7. Ca^2+^ Inhibitory Confirmation

Once in the preliminary experiments, the plant extract relaxant effects against high K^+^ were observed, further confirmation of Ca^2+^ channel blockade (CCB) was determined by making tissues Ca^2+^ free by 45 min incubation in Ca^2+^-free Tyrode’s solution with a chelating agent (EDTA, 0.1 mM). To further deplete the intracellular stores of Ca^2+^, Tyrode solution without Ca^2+^ was replaced with K^+^-rich and Ca^2+^-free Tyrode’s solution with the following concentrations (mM): potassium chloride: 50, sodium chloride: 91.03, sodium dihydrogen phosphate dehydrates: 0.32, sodium bicarbonate: 11.9, magnesium chloride hexahydrate: 0.50, glucose: 5.05. At the end of 45 min incubation period of ileal tissue in this solution, cumulative Ca^2+^ CRCs in the absence and presence of increasing concentrations of *G*. *tenax* were constructed by adding exogenous CaCl_2_ in the bath, and the obtained CRCs of the plant and/or papaverine was compared with the standard CCB agent, verapamil [30].

### 4.8. PDE Inhibitory Confirmation

The relaxant effect of *G*. *tenax* against CCh and high K^+^ at similar potencies is an indication of PDE inhibition [45]; therefore, indirect confirmation for PDE inhibition by *G*. *tenax* was evaluated by constructing dose-mediated inhibitory CRCs of isoprenaline against CCh in the absence (control) and presence of *G*. *tenax*. A leftward shift in the inhibitory curves indicates potentiation and is considered the plant/test material inhibitory potential on PDE [46].

### 4.9. Statistical Analysis

The obtained results are mentioned as the mean *±* standard error of the mean (SEM), and “*n*” represents the number of the experiment. The examination of median effective concentration (EC50) was done with a 95% (CI) Confidence interval. The multiple comparisons of CRCs with respective controls were done by applying test mainly Student’s test or two-way ANOVA followed by Bonferroni’s post-test. Additionally, the Chi-square (χ^2^) test was used to evaluate protection from diarrhea, and a comparison was made between all groups and the saline control group. *p* < 0.05 was considered statistically significant. For regression analysis, GraphPad Prism (Version 4) was employed.

## 5. Conclusions

The methanolic extract of *G*. *tenax* protected mice in a dose-mediated way from castor oil-induced diarrhea and also showed concentration-dependent anti-spasmodic effects tested in isolated rat ileum. The spasmolytic effect observed against both CCh and high K^+^ at similar concentrations indicates dual inhibitory effects of *G*. *tenax* against PDE enzymes and Ca^2+^ channels, similar to papaverine. The PDE inhibition was further confirmed by the leftward shift of isoprenaline inhibitory CRCs, whereas the rightward deflection of Ca^2+^ CRCs towards the right authenticated the CCB-like effect of *G*. *tenax*. GC-MS analysis shows the identification of 28 phytoconstituents in the extract of *G*. *tenax* fruits where 6,6-dideutero-nonen-1-ol-3 (20.98%) was recorded as the main constituent and 3-Deoxy-d-mannoic lactone (15.36%) as a second major constituent in addition to the methyl furfural, pyranone, carboxylic acid, vitamin E, fatty acid ester, hydrocarbon, steroids, sesquiterpenes, phytosterols, and ketones. Thus, this study provides a rationale for the medicinal use of *G*. *tenax* fruits in addressing gastrointestinal hyperactivity-related disorders with detailed pharmacodynamics explored. We recommend further in-depth molecular assays to develop *G*. *tenax* for clinical use in the future.

## Figures and Tables

**Table 1:** List of phytoconstituents present in methanolic extract of *G. tenax* fruits.

S. No.	Compound Name	RT (Min)	% Area	Nature of Compound
1	1,2,3-Propanetriol	5.420	11.40	Phenol
2	6-Amino-5-nitroso-1H-pyrimidine-2,4-dione	5.974	4.31	Pyrimidinediones
3	4H-Pyran-4-one, 2,3-dihydro-3,5-dihydroxy-6-methyl	6.821	8.27	Pyranone
4	2-Pentanone	7.976	1.85	Ketone
5	5-Hydroxymethylfurfural	8.429	11.01	Hydroxymethylfurfural
6	1-Tetradecene	10.230	0.11	Hydrocarbon
7	1,2-Cyclobutanedicarboxylic acid	11.427	5.72	Carboxylic acid
8	6,6-Dideutero-nonen-1-ol-3	12.334	20.98	Alcohol
9	3-Deoxy-d-mannoic lactone	14.484	15.36	Lactone
10	Hexadecanoic acid, methyl ester	16.473	0.62	Fatty Acid Ester
11	Hexadecanoic acid, ethyl ester	17.138	0.23	Fatty Acid Ester
12	9,12-Octadecadienoic acid (Z,Z)-, methyl ester	18.114	1.50	Fatty Acid Ester
13	9-Octadecenoic acid, methyl ester, (E)-	18.172	1.84	Fatty Acid Ester
14	Octadecenoic acid, methyl ester	18.408	0.49	Fatty Acid Ester
15	Ethyl (9Z,12Z)-9,12-octadecadienoate	18.721	0.17	Fatty Acid
16	Glycidyl palmitate	19.950	0.44	Fatty Acid Ester
17	1,8,11-Heptadecatriene, (Z,Z)-	21.395	0.87	Hydrocarbon
18	Glycidyl oleate	21.430	1.20	Ester
19	15-Hydroxypentadecanoic acid	21.629	0.43	Fatty Acid
20	9,12-Octadecadienoic acid (Z,Z)-, 2-hydroxyl-1-(hydroxymethyl)ethyl ester	23.19	2.05	Fatty Acid Ester
21	9-Octadecenamide, (Z)-	23.880	0.56	Fatty Acid Primary amide
22	Squalene	24.021	1.02	Hydrocarbon
23	2H-1-benzopyran-6-OL, 4-dihydro-2,5,7,8-tetramethyl-2-(4,8,12-trimethyltridecyl)	27.846	3.79	Vitamin E acetate
24	Beta-sitosterol	30.127	2.12	Phytosterol
25	4,4-Dimethyl-5.alpha.-D1-androstane-3.beta	31.105	1.02	Acetate
26	Ergost-5-en-3-ol,(3.beta.)	31.406	1.54	Steroid
27	Stigmasta-4,22-dien-3-one	33.005	0.32	Steroid
28	1,1,6-Trimethyl-3-methylene-2-(3,6,10,13,14–pentamethyl-3-ethenyl-pendadec-4-enye)cyclohexane	33.450	0.35	Sesquiterpenoid
			99.66	

**Table 2:** Antidiarrheal activity of the methanolic extract of *G*. *tenax* fruits against castor oil (10 mL/kg)-evoked diarrhea in mice.

Treatment (p.o.), Dose (mg/kg)	No. of Mice with Diarrhea	% Protection
Saline (10 mL/kg) + Castor oil	5/5	0
*G*. *tenax* (200 mg/kg) + Castor oil	3 */5	40
*G*. *tenax* (400 mg/kg) + Castor oil	1 */5	80
Loperamide (10 mg/kg) + Castor oil	0 **/5	100

* *p* < 0.05 and ** *p* < 0.01 vs. Saline + Castor oil treated group (χ^2^-test).

## Data Availability

Not Applicable.

## Sample Availability

Samples of the compounds are available from the authors.
